# A Novel Signature of Necroptosis-Associated Genes as a Potential Prognostic Tool for Head and Neck Squamous Cell Carcinoma

**DOI:** 10.3389/fgene.2022.907985

**Published:** 2022-06-09

**Authors:** Jing Huang, Hongqi Huo, Rong Lu

**Affiliations:** ^1^ Department of Pharmacy, Fujian Cancer Hospital, Fujian Medical University Cancer Hospital, Fuzhou, China; ^2^ Nuclear Medicine Department, Handan Central Hospital, Handan, China; ^3^ Department of Laboratory Medicine, The First Affiliated Hospital of Xiamen University, Xiamen Key Laboratory of Genetic Testing, School of Medicine, Xiamen University, Xiamen, China

**Keywords:** risk score, prognosis, squamous cell carcinoma, necroptosis, immune, tumor

## Abstract

**Background:** Head and neck squamous cell carcinoma (HNSCC) arises from squamous cells in the oral cavity, pharynx and larynx. Although HNSCC is sensitive to radiotherapy, patient prognosis is poor. Necroptosis is a novel programmed form of necrotic cell death. The prognostic value of necroptosis-associated gene expression in HNSCC has not been explored.

**Material and Methods:** We downloaded mRNA expression data of HNSCC patients from TCGA databases and Gene Expression Omnibus (GEO) databases, and compared gene expression between tumor tissues and adjacent normal tissues to identify differentially expressed genes (DEGs) and necroptosis-related prognostic genes. A model with necroptosis-related genes was established to predict patient prognosis *via* LASSO method and Kaplan-Meier analysis. GSE65858 data set (*n* = 270) from GEO was used to verify the model’s predictive ability. Gene set enrichment analyses, immune microenvironment analysis, principal component analysis, and anti-tumor compound IC_50_ prediction were also performed.

**Results:** We identified 49 DEGs and found 10 DEGs were associated with patient survival (*p* < 0.05). A risk model of 6-gene signature was constructed using the TCGA training data set and further validated with the GEO data set. Patients in the low-risk group survived longer than those in the high-risk group (*p* < 0.05) in the GEO validation sets. Functional analysis showed the two patient groups were associated with distinct immunity conditions and IC_50_.

**Conclusion:** We constructed a prognostic model with 6 necroptosis-associated genes for HNSCC. The model has potential usage to guide treatment because survival was different between the two groups.

## Introduction

Head and neck squamous cell carcinomas (HNSCC) arise from squamous cells in oral cavity, pharynx, and larynx. Although HNSCC is sensitive to radiotherapy, patient prognosis is poor. The most common risk factors for HNSCC include smoking, alcohol consumption and human papilloma virus infection (Fakhry et al., 2008). The treatment of HNSCC is integrated and multimodal including surgery, radiotherapy, chemotherapy, and most recently immonotherapy. Patients with HNSCC still suffer from poor survival in spite of the progress in new treatment strategies. To improve patient survival, new prognostic models are needed for precision medicine in addition to identification of novel therapeutic targets.

Necroptosis is another mode of regulated cell death mimicking apoptosis and necrosis. Necroptosis is associated with a range of pathological conditions and diseases, including cancer. It is mediated by Fas, TNF, and LPS and death receptors (Vanden Berghe et al., 2014). Binding of ligands and rceptors activates Receptor-interacting protein kinase-3 (RIP3 or RIPK3), which phosphorylates and activates mixed lineage kinase domain-like (MLKL) (Sun et al., 1999). Phosphorylated MLKL translocates to cellular membranes, and ruptures cellular membranes, leading to cell swelling and release of intracellular components (Dondelinger et al., 2014; Hildebrand et al., 2014; Wang et al., 2014). Another characteristic of necroptosis is inhibition of caspase-8.

A plethora of evidences has shown that necroptosis is associated to tumors. Downregulation of RIPK3 was reported in several cancers. For example, RIP3 expression was reduced in breast cancer (Koo et al., 2015). RIP3 expression was also decreased in colorectal cancer and was an independent prognostic factor of survival. Overexpression of RIP3 proliferation of colorectal cancer cells *in vitro* (Feng et al., 2015). In acute myeloid leukemia, RIP3 expression was again reduced in most samples. In DA1-3b leukemia cells, overexpression of RIP3 induced necroptosis (Nugues et al., 2014). Li et al. (2020) reported that necroptosis was associated with survival of HNSCC patients (Li et al., 2020). Although necroptosis plays an important role in patient survival of a variety of tumors, the role of necroptosis in HNSCC remains unclear.

The present study aimed to explore the potential roles of necroptosis-related genes on the survival of HNSCC patients and developed a risk-score model with necroptosis-related gene expression levels. The results might also further our understanding of necroptosis in HNSCC.

## Materials and Methods

### Database

The Cancer Genome Atlas (TCGA) (https://portal.gdc.cancer.gov/repository) is a public funded project that aims to discover major cancer-causing genomic alterations. In the present study RNA sequencing (RNA-seq) data of tumor tissues of 487 HNSCC patients and 42 matched normal tissues was downloaded from TCGA database. Specifically expression data of 67 necroptosis-associated genes was used for analysis (Supplementary Table S1). An independent data set was downloaded from the GEO database (GSE65858, *n* = 270) (https://www.ncbi.nlm.nih.gov/geo/) to validate the prognostic model based on TCGA database.

Our study did not require ethical approval because we used information freely available in the public domain.

### Identification of Different Expression Genes

Expression of necroptosis-related genes between tumor tissues and adjacent normal tissues was compared to identify DEGs by using the “linear models for microarray data (limma)” R package available from the Bioconductor project (www.bioconductor.org). Limma is a popular package for analyzing microarray and RNA-seq data. We calculated the ratios for all genes between samples to determine the fold-change (FC) in expression between groups. DEGs were defined as |log2FC|>1. The false discovery rate (FDR) was set to be below 0.05.

A protein-protein interaction (PPI) network of DEGs were developed utilizing the STRING database (https://string-db.org/). The STRING database integrates all known and predicted associations between proteins, including both physical interactions as well as functional associations. The Gene Ontology (GO) was used to describe the biological processes (BPs) and molecular functions (MFs) of the necroptosis-related genes. The Database for Annotation, Visualization and Integrated Discovery (DAVID) was used to perform functional annotation mapping of genes. Kyoto Encyclopedia of Genes and Genomes (KEGG) was used to brose genome maps (Huang et al., 2007). R-package ‘GOplot’ was used to provide a deeper insight into expression data and generate plots.

### Risk Signature Establishment

Patients’ clinical data was downloaded from TCGA. The univariate Cox proportional hazard regression analysis was used for screening of prognostic genes. Least absolute shrinkage and selection operator (Lasso) regression was conducted with 10-fold cross-validation and a *p* value of 0.05.

After identification of potential prognostic genes, the risk score was calculated as follow:

$$\text{risk score}=\overset{n}{\underset{i}{\sum }}Xi*Yi$$ (1)

Where X is coefficient and Y is gene expression level.

Using the median risk score as the cutoff point, we divided the patients into either the low-risk group or the high-risk group (Meng et al., 2019; Hong et al., 2020). Overall survival (OS) was evaluated by Kaplan-Meier method using the log-rank test to find difference between the two groups. We performed Principal Component Analysis (PCA) in R package using the prcomp function. T-distributed stochastic neighbor embedding (t-SNE) was used for data exploration and embedding high-dimensional data for visualization. The 1-, 3-, and 5-year time-dependent receiver operating characteristics (ROC) curves were plotted in R package using the “survival,” “survminer” and “timeROC” functions. We used univariate Cox regression and multivariable Cox regression to explore whether patient and tumor characteristics (age, gender, grade of tumor differentiation, and TNM stage) and the calculated risk scores were independent risk factors. The rms package was employed to fit regression model and depict nomograms for the 1-, 3-, and 5-year OS.

The risk score model was validated with the HNSCC GEO dataset (GSE65858).

### Gene Set Enrichment Analyses

Genes that might have an association with the development of HNSCC were identified by GSEA. GSEA was performed by using the fast preranked gene set enrichment analysis (fgsea) package. KEGG gene sets in GSEA database were downloaded. A FDR value < 0.05 was considered to be statistically significant.

### Analysis of Tumor Microenvironment and Immune Checkpoints

The CIBERSORT, EPIC, MCPcounter, QUANTISEQ, TIMER, and XCELL algorithms were used to assign cell types of TME of the patients and analyze the immunological characteristics (http://timer.cistrome.org/) (Hong et al., 2020). TME scores and immune checkpoint activation were compared between the two groups with ggpubr R package.

### Prediction of Treatment Response

pRRophetic R package was used for prediction of drug response as indicated by half-maximal inhibitory concentration (IC_50_) according to Genomics of Drug Sensitivity in Cancer (GDSC) (Geeleher et al., 2014).

## Results

### Identification of Different Expression Genes and Functional Analysis

**FIGURE 1:**
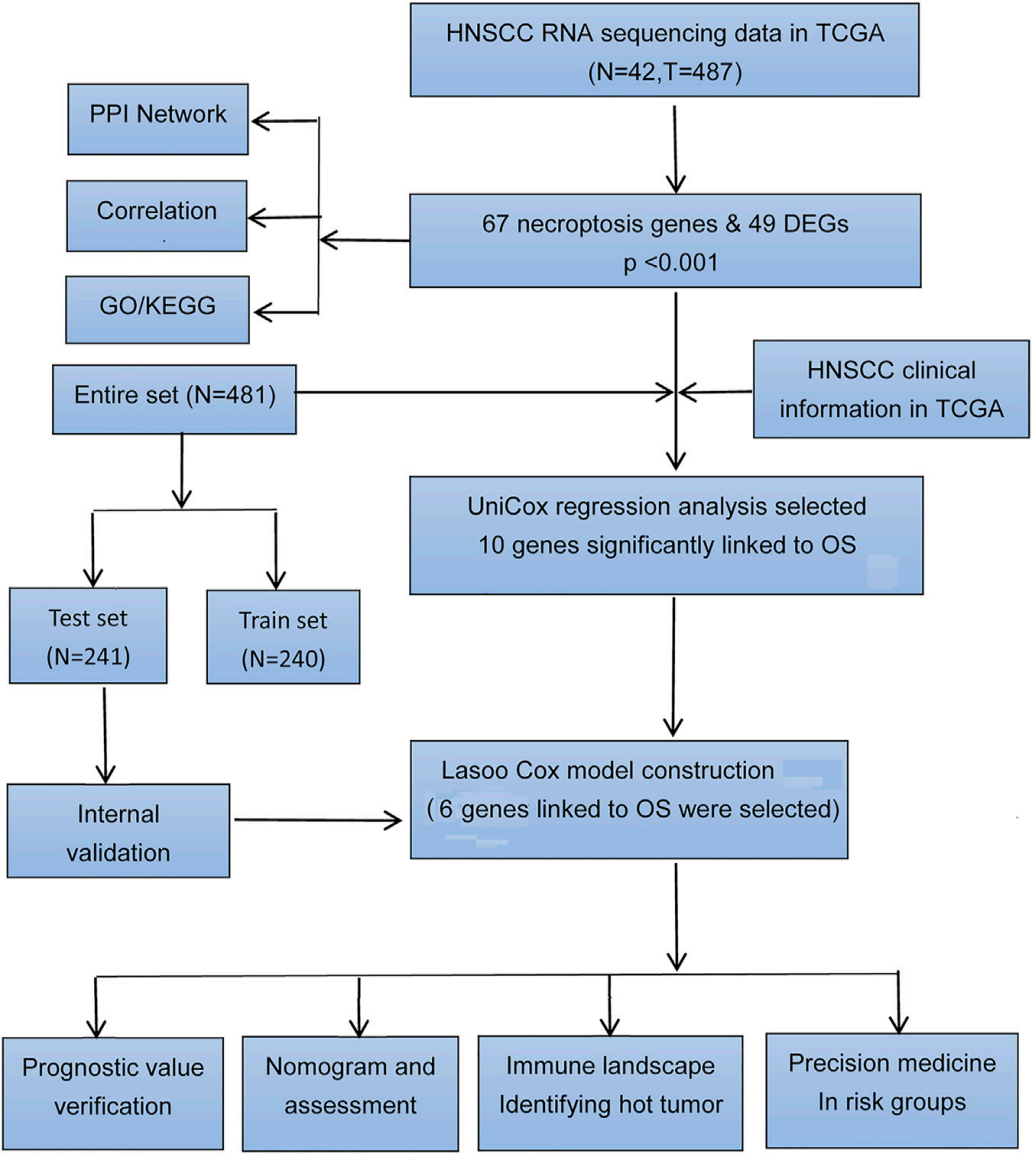
Flow diagram of the study.

**FIGURE 2:**
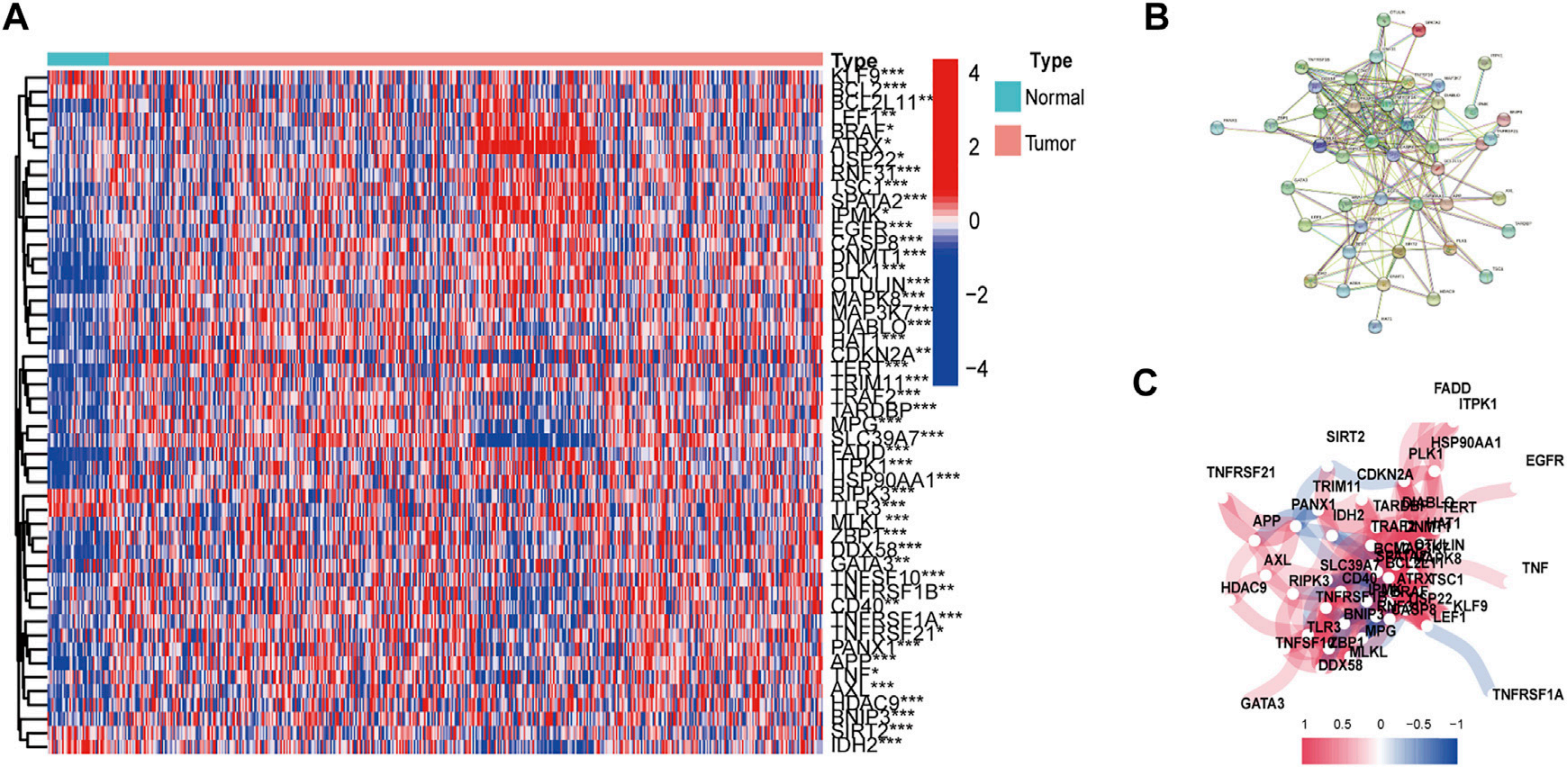
Expressions and interactions of 67 necroptosis-related genes in the normal and tumor tissues. **(A)** Heatmap of necroptosis-related genes. **(B)** Gene interactions revealed by PPI. **(C)** Gene correlation network (Red and blue lines indicate positive and negative association respectively). *p < 0.05; **p < 0.01; ***p < 0.001, tumor vs. normal tissues.

The workflow of DEG identificatin is shown in Figure 1. We compared the expression levels of 67 necroptosis-related genes between 487 tumor samples and 42 adjacent normal samples from the HNSCC patients and identified 49 DEGs (*p* < 0.05). Necroptosis-related genes are listed in Supplementary Table S1. Among 49 DEGs, 42 were upregulated and 7 were downregulated in the tumor tissues (Figure 2A). Interactions of the 49 DEGs were revealed by PPI analysis (Figure 2B). Figure 2C shows the associations between the necroptosis-related genes.

**FIGURE 3:**
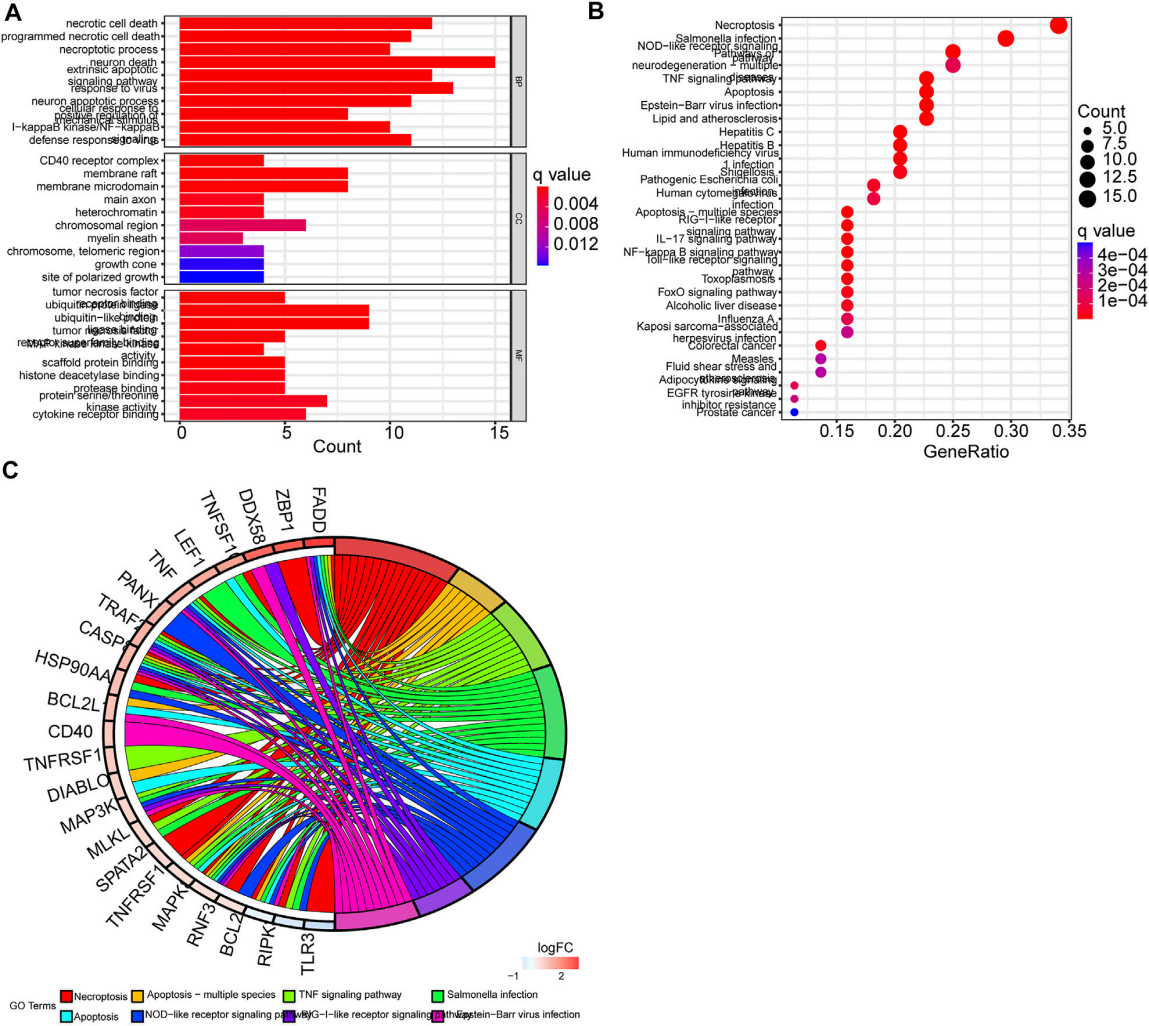
Functional analysis of differentially expressed genes (DEGs). **(A)** GO enrichment barplot (longer bars indicate more enriched genes, and redness means the differences between the low-risk and the high-risk groups). **(B)** KEGG pathway bubble graph (bigger bubbles mean more enriched genes, and redness means the differences between the low-risk and the high-risk groups; q-values were adjusted *p*-values). **(C)** Circular plot of KEGG pathways. Left: Downregulation gene expression is indicated by green color and upregulation gene expression by red color. Right: Genes are linked *via* color ribbons to their function (*p* < 0.05 for all).

GO enrichment analysis and KEGG pathway analysis showed that these DEGs were related to extrinsic apoptotic signaling pathway, TNF signaling pathway, necroptosis, and apoptosis (Figures 3A–C) (Supplementary Table S2).

### Model Construction and Validation

**FIGURE 4:**
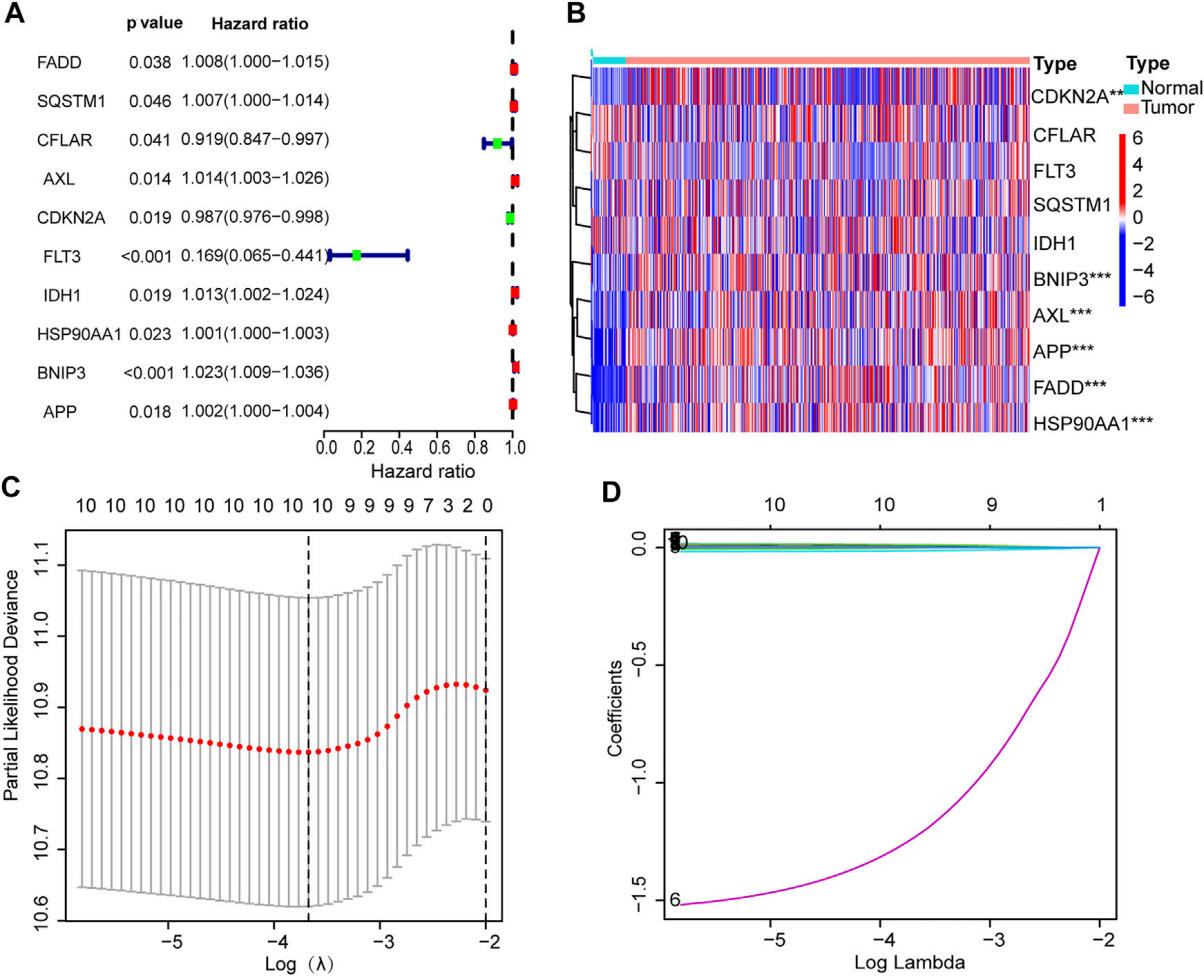
Establishment of prognostic signature. **(A)** Ten prognostic genes with *p* values. **(B)** Heat map of gene expression. **(C)** LASSO analysis with 10-fold cross-validation identified 6 prognostic genes. **(D)** Coefficient profile plots of 6 prognostic genes.

Univariate Cox regression analysis showed that six necroptosis-related genes were correlated with OS (*p* < 0.05 for all) (Figure 4A). Then we constructed a six-gene prognostic signature by LASSO regression analysis (Figures 4B–D). Results of the Lasso regression showed the model had good predictive performances. Figure 4 shows the first-rank value of Log (λ). The six genes are *Fas-associated protein with death domain* (*FADD*), *Anexelekto* (*AXL*), *heat shock protein 90-alpha* (*HSP90AA1*), *BCL2 Interacting Protein 3* (*BNIP3*), *amyloid precursor protein* (*APP*), *and cyclin-dependent kinase inhibitor 2A* (*CDKN2A*)*.*

The risk score formula was set up as follow (Hong et al., 2020): risk score = FADD × (0.0037) + AXL × (0.0131) + CDKN2A × (−0.0105) + HSP90AA1 × (0.0009) + BNIP3 × (0.0201) + APP × (0.0009).

**FIGURE 5:**
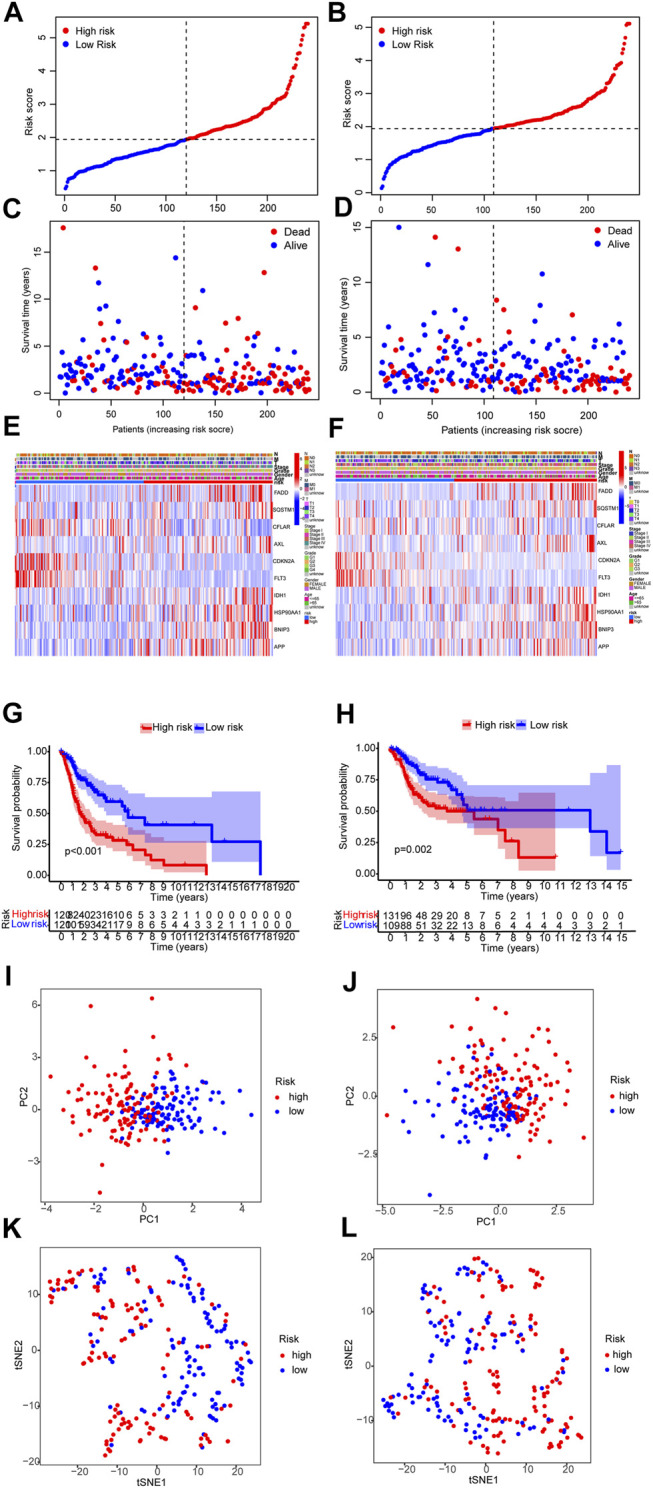
Prognosis assay in the training and validation sets. **(A,B)** Risk score of the training and validation sets. **(C,D)** Survival comparison of the low-risk and the high-risk groups in the training and validation sets. **(E,F)** The heat map of clinicopathological characteristics and expression of 6 genes. **(G,H)** Survival curves of the low-risk and the high-risk groups in the training and validation sets. **(I–L)** PCA and T-NSE plots of the low-risk and the high-risk groups of the training and validation sets.

The distribution of risk scores and survival times were compared between the low-risk group and the high-risk group. Figure 5 shows the high-risk group had the worse prognoses in both training and validation sets.

### Construction of Nomogram

**FIGURE 6:**
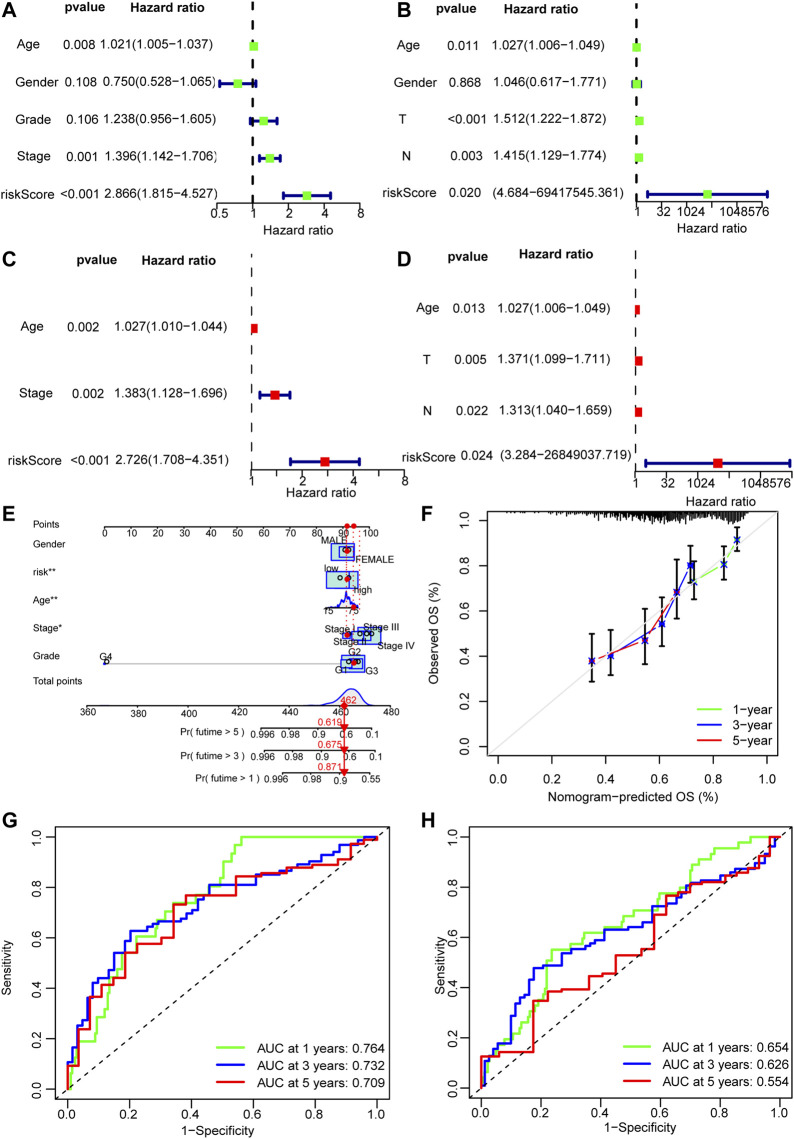
Nomogram of the model. **(A–D)** Univariate and multivariate Cox analyses of clinicopathological characteristics and risk score with overall survival (OS) in the training and validation sets. **(E)** The nomogram that integrated the risk score, age, and tumor stage to predict OS. **(F)** The calibration curves for OS. **(G,H)** The ROC curves of the low-risk and the high-risk groups of the training and validation sets.

A nomogram was established on the basis of the results of the multivariate analysis in the development cohort, which identified risk score, age, tumor grade, and tumor stage to be independent prognostic factors. The hazard ratios (HR) of these factors are shown in Figures 6A–E.

### Assessment of Risk Model

The discriminative power of whether the constructed nomogram could correctly predict the probability of OS was quantified using the area under the time-dependent ROC curves (AUC). In the training set, 1-, 3-, and 5-year AUCs were 0.639,0.653 and 0.582 respectively; and they were 0.651,0.586, and 0.597 respectively in the validation set (Figures 6F,G).

### Gene Set Enrichment Analysis

**FIGURE 7:**
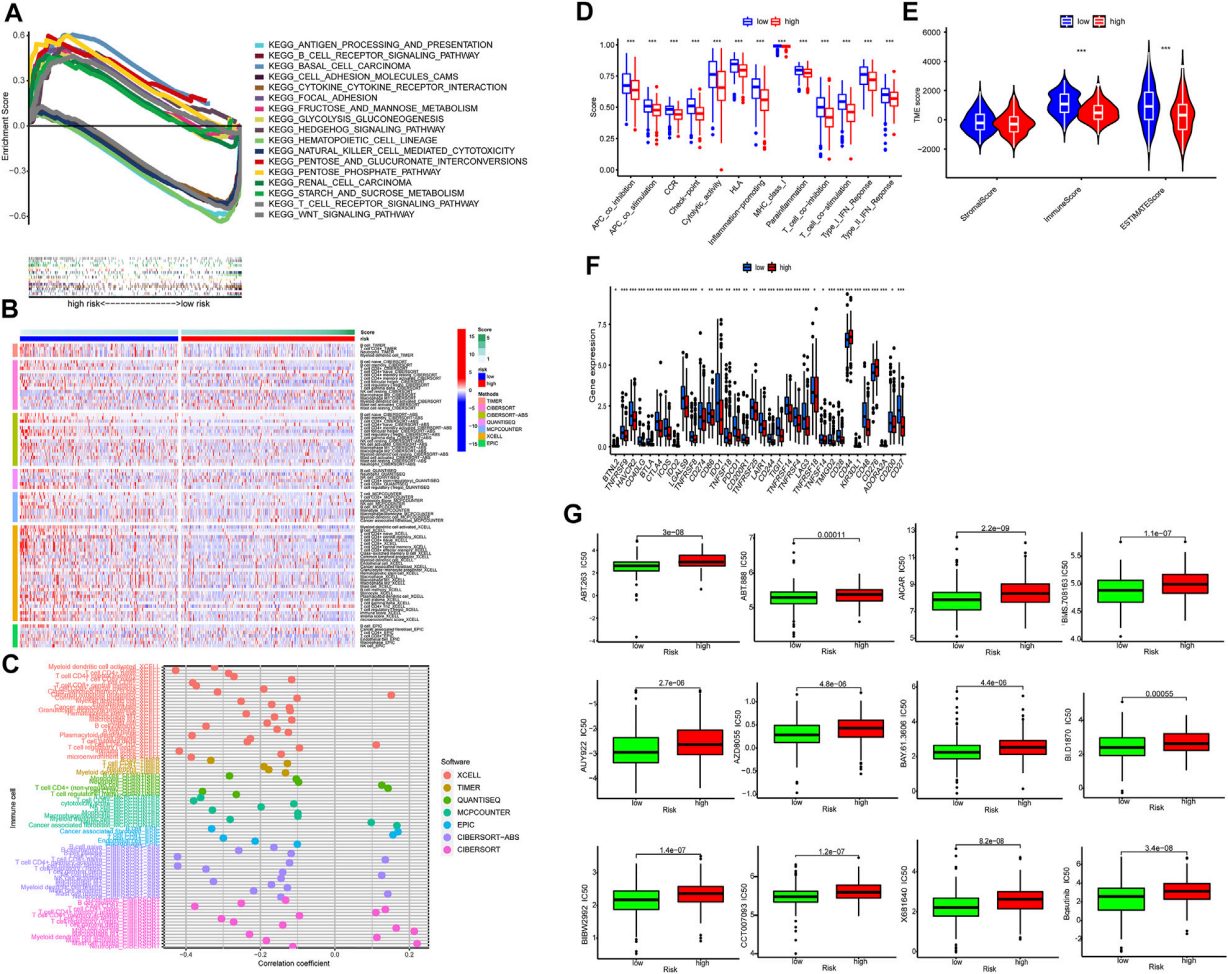
Tumor immune microenvironment. **(A)** Top 10 pathways by GSEA analysis. **(B,C)** The heat maps and bubble chart of immune cells. **(D)** The ssGSEA scores of immune functions of the low-risk group and the high-risk group. **(E)** The comparison of immune and stromal scores between the low-risk group and the high-risk group. **(F)** The expression of 34 checkpoints of the low-risk group and the high-risk group. **(G)** Twelve targeted and immunotherapeutic drugs with significantly lower IC_50_ for the low-risk group compared to the high-risk groups.

GSEA identified 20 enriched pathways (Supplementary Table S3). The top pathways included basal cell carcinoma, pentose phosphate pathway, hedgehog signaling pathway, pentose phosphate pathway, small cell lung cancer, focal adhesion, extracellular matrix–receptor (ECM-receptor) interaction pathway, TGF-beta signaling pathway, renal cell carcinoma, glycolysis gluconeogenesis, and WNT signaling pathway. Most of them are associated to tumor development and metastasis and were enriched in the high-risk group. On the other hand, seven pathways enriched in the low-risk group were related to immunity, such as transendothelial migration of leukocyte and NK cell-mediated cytotoxicity (Figure 7A). Therefore the two groups had distinct pathways.

### Cold and Hot Tumors

Immunotherapy is promising for solid tumors. Immune cell infiltrates are crucial for host immune reaction. The infiltration of immune cells was estimated by several algorithms. As shown in Figures 7B,C, more immune cells were observed in the low-risk group, including naïve B cells, memory B cells, CD8^+^ T cells, naive CD4^+^ T cells, memory resting CD4^+^ T cells, activated dendritic cell and activated mast cells (Supplementary Table S4). In addition, all of the 13 immune-related pathways had higher activity in the low-risk group (Figure 7D).

Figures 7D,E show both immune score and microenvironment score were higher in the low-risk group. Besides, the immune checkpoint (IC) expression, including CTLA-4, PD-1, LAG-3, and TIGIT was lower in the low-risk group (Figure 7F). This indicated that tumors of the low-risk group were more sensitive to immunotherapy (hot tumor) and tumors of the high-risk group were more resistant to immunotherapy (cold tumor) (Figure 7G).

Finally, we found IC_50_ of the anti-tumor compounds was usually lower in the low-risk group, such as Bosutinib, a dual kinase inhibitor of both the BCR-ABL and Src tyrosine kinases.

## Discussion

We assessed the prognostic value of expression of 10 necroptosis-related genes in a retrospective analysis of 487 patients with HNSCC in TCGA database. Our results show that HNSCC patients could be groups into either high-risk group or low-risk group by a calculated score based on the 6 genes. Patients in the high-risk group were associated with worse OS.

Among 6 necroptosis-related genes, five are associated with a higher risk of HNSCC. They include *FADD*, *AXL*, *HSP90AA1*, *BNIP3*, and *APP* genes. FADD is an adaptor protein that noncovalently associates with the death domain of the cytoplasmic region of Fas (Chinnaiyan et al., 1995). FADD can promote survival of osteosarcoma cells (Hollomon et al., 2020). AXL is a receptor tyrosine kinase, that is, activated by Gas6. AXL promotes epithelial-to-mesenchymal transition and cancer progression. It is also associated with anti-tumor drug resistance (Wu et al., 2014; Gay et al., 2017; Boshuizen et al., 2018). AXL was supposed to be a target of cancer therapy (Dang et al., 2009; Mullen et al., 2011). HSP90AA1 is a highly conserved molecular chaperon in evolution (Condelli et al., 2019). HSP90AA1 is expressed in tumors and can activates many oncogenic proteins, thereby stimulating cancer cell survival, proliferaton, and invasiveness (Eustace et al., 2004; Chehab et al., 2015). HSP90AA1 is also a potential molecular target in cancer therapy.

BNIP3 is a stress sensor protein (Bruick, 2000). BNIP3 interacts with anti-apoptotic proteins, including E1B 19 kDa protein and Bcl2. BNIP3 was found to play an important role in carcinogenesis (Vijayalingam et al., 2010). BNIP3 expression is upregulated by hypoxia and in a variety of cancers (Sowter et al., 2003; Giatromanolaki et al., 2004; Leo et al., 2006; Shaida et al., 2008; Burton and Gibson, 2009). APP is most abundant in neurons and abnormal processing of APP is associated to senile dementia. Surprisingly several cancers have abnormal APP expression (Takagi et al., 2013). Lim et al. (2014) reported that APP knockdown reduced breast cancer cell growth (Lim et al., 2014).

The only gene that was associated with low risk of death was *CDKN2A*. CDKN2A encodes for tumor suppressor protein p16^INK4a^, which is often inactivated in cancer (Witcher and Emerson, 2009) (Alevizos et al., 2012). Veganzones et al. (2015) reported that approximately 90% of HPV-negative HNSCC tumors exhibited low expression of DKN2AC, mainly due to gmutations, loss of heterozygosity, and hyper-methylation of the gene promoter (Veganzones et al., 2015). In addition, CDKN2A loss-of-function in patients with non-small cell lung cancer had inferior OS when treated with immune checkpoint blockade (ICB) when compared to wild-type patients (Gutiontov et al., 2021).

Immune checkpoints (ICs) ensure the maintenance of immune homeostasis by regulating the time course and intensity of immune responses. However, receptor-based signaling cascades from ICs play a negative regulatory role in T cells, allowing tumors to evade immune surveillance by inducing immune tolerance. Immune checkpoint inhibitors (ICIs) block checkpoint proteins from binding with their partner proteins, allowing T cells to kill cancer cells. Immune checkpoint inhibitors are approved to treat a variety of cancer types, including head and neck cancer. Human tumors evade immune attack in tumor tissue and different immune profiles predict different outcomes of immunotherapy (Hui and Chen, 2015; Bonaventura et al., 2019; Pérez-Romero et al., 2020). Effective anti-PD-1/-L1 therapy depends on lymphocyte infiltration (“hot tumors”). In contrast, “cold tumors” lacks T cells infiltration. Pembrolizumab and nivolumab that target PD-1 could improve OS of patients with recurrent HNSCC(Ferris et al., 2016; Seiwert et al., 2016). We found patients in the high-risk group were characterized by higher expression of ICs, suggesting that necroptosis-related gene expression might guide immunotherapy. We also found IC_50_ of several anti-tumor agents was lower in the low-risk groups. For example, Bosutinib, a dual kinase inhibitor of both the BCR-ABL and Src tyrosine kinases and is used in the therapy of Philadelphia chromosome-positive chronic myelogenous leukemia, has lower IC_50_ in the low-risk group.

By our model, we found pathways such as focal adhesion, basal cell carcinoma, hedgehog signaling and WNT signaling (Kumar et al., 2021) were enriched in the high-risk group. These pathways are associated with tumor development. In the low-risk group, most of the enriched pathways were related to immunity, including leukocyte transendothelial migration and NK cell-mediated cytotoxicity. These may explain why the local immune signatures are different between the two groups, which might predict prognoses and response to immunotherapy.

Our study has some limitations. First, this was a retrospective study based on databases, which may have some inherent biases. Second, although our nomogram was externally validated, the validation database contained a small sample size. Third, the conclusion needs to be validated using other populations.

In conclusion, we established a novel necroptosis-associated gene signature for prognosis of HNSCC. The established signatures reflected that necroptosis would be associated with responses to targeted therapy and immunotherapy of HNSCC. However, our study was susceptible to the inherent biases of the retrospective study. The potential of this signature in predicting patient survival and treatment responses need to be validated in future tests.

## Data Availability

The original contributions presented in the study are included in the article/Supplementary Material, further inquiries can be directed to the corresponding authors.

## Supplementary Material

Supplementary Table S1 The table of necroptosis-related genes refers to GSEA and previous reports.

Supplementary Table S4 The profile of significantly differently infiltrated immune cells in the high- and low-risk groups based on different platforms.
