# Supplementary material for: A Novel Signature of Necroptosis-Associated Genes as a Potential Prognostic Tool for Head and Neck Squamous Cell Carcinoma
Source: Front Genet. 2022 Jun 9;13:907985. doi: 10.3389/fgene.2022.907985 (PMC9218670; doi:10.3389/fgene.2022.907985)
Supplement: Supplementary file 2 [file Table2.DOCX]

Tables S2.Differentially expressed genes (DEGs) Between normal and tumor tissues in HNSCC patients

| **gene** | **conMean** | **treatMean** | **logFC** | **pValue** |
| --- | --- | --- | --- | --- |
| FADD | 3.963397905 | 16.85389955 | 2.088272713 | 2.12E-20 |
| MLKL | 2.197850448 | 3.017474055 | 0.457248145 | 0.000460015 |
| RIPK3 | 2.927709683 | 2.247838079 | -0.381234385 | 0.000493829 |
| TLR3 | 2.312732207 | 1.513987123 | -0.61124529 | 1.68E-05 |
| TNF | 1.357543384 | 2.679604772 | 0.981021923 | 0.02638549 |
| TSC1 | 2.42223081 | 3.414212294 | 0.495216424 | 5.46E-08 |
| TRIM11 | 2.693334619 | 4.211505142 | 0.644942446 | 1.43E-10 |
| CASP8 | 2.179193281 | 3.886265177 | 0.834590185 | 9.21E-11 |
| ZBP1 | 0.240689845 | 0.858759478 | 1.835078844 | 1.96E-08 |
| MAPK8 | 2.222214881 | 2.888366739 | 0.378255607 | 1.94E-05 |
| IPMK | 2.53475226 | 3.201310373 | 0.336817807 | 0.017145695 |
| ITPK1 | 6.118260119 | 10.5909695 | 0.79164131 | 1.26E-12 |
| TNFRSF1A | 36.45165071 | 48.28559273 | 0.405608639 | 8.18E-08 |
| TNFSF10 | 36.37988676 | 78.21772112 | 1.104354451 | 2.51E-06 |
| TNFRSF1B | 7.346338667 | 11.20445584 | 0.608975273 | 0.003002394 |
| TRAF2 | 5.089769881 | 9.570001234 | 0.91091868 | 8.16E-09 |
| PANX1 | 8.559248262 | 16.45352847 | 0.942841006 | 1.51E-09 |
| OTULIN | 2.685578219 | 5.093310179 | 0.923370837 | 1.99E-17 |
| USP22 | 21.15855793 | 25.29837318 | 0.257803312 | 0.011584587 |
| MAP3K7 | 4.576259262 | 6.534322604 | 0.513868895 | 1.27E-12 |
| DIABLO | 0.944383169 | 1.370470936 | 0.537227498 | 5.11E-11 |
| DNMT1 | 5.536189405 | 12.6947823 | 1.197270448 | 1.55E-16 |
| BRAF | 1.95457719 | 2.409631684 | 0.301956084 | 0.022518096 |
| AXL | 4.577396952 | 11.21429173 | 1.292739193 | 5.89E-08 |
| CDKN2A | 1.082880024 | 10.12294572 | 3.224683851 | 0.002634294 |
| BCL2 | 1.655396167 | 1.519401894 | -0.123672997 | 0.000297232 |
| HAT1 | 5.199461214 | 8.26679176 | 0.668965412 | 1.39E-13 |
| SIRT2 | 12.86129617 | 8.649717285 | -0.572311161 | 0.000136489 |
| PLK1 | 3.61625961 | 13.20515956 | 1.868531577 | 3.88E-21 |
| MPG | 11.60727824 | 15.03834311 | 0.373615905 | 2.58E-05 |
| GATA3 | 2.853622337 | 1.949851757 | -0.54942997 | 0.001045947 |
| ATRX | 2.938952167 | 4.122059138 | 0.488063325 | 0.032311984 |
| TERT | 0.020884503 | 0.156301007 | 2.903822358 | 3.75E-15 |
| SLC39A7 | 35.99593762 | 51.17965829 | 0.507736417 | 9.38E-06 |
| SPATA2 | 3.582508738 | 4.866302867 | 0.441855889 | 4.60E-07 |
| RNF31 | 3.690097069 | 4.706704018 | 0.351058364 | 0.000431785 |
| IDH2 | 95.38033881 | 54.63913616 | -0.803757233 | 5.33E-08 |
| KLF9 | 11.61176069 | 7.137167456 | -0.702163217 | 0.000674075 |
| HDAC9 | 0.462962297 | 0.710675331 | 0.618295915 | 0.000985265 |
| HSP90AA1 | 153.2994448 | 257.3463229 | 0.747358697 | 4.03E-12 |
| LEF1 | 1.51955286 | 3.093129831 | 1.025420529 | 0.001015187 |
| BNIP3 | 7.872902548 | 13.34210886 | 0.761019193 | 5.50E-05 |
| CD40 | 6.649511571 | 10.5263945 | 0.66269109 | 0.001505053 |
| BCL2L11 | 3.256506476 | 5.373883832 | 0.722640039 | 4.78E-09 |
| EGFR | 15.94821867 | 45.10009896 | 1.499735308 | 6.47E-08 |
| DDX58 | 4.187148048 | 13.62415014 | 1.702126404 | 6.11E-13 |
| TARDBP | 13.52158786 | 18.51776327 | 0.453645269 | 2.88E-12 |
| APP | 68.83352405 | 150.5583971 | 1.129139896 | 6.91E-14 |
| TNFRSF21 | 42.75331205 | 55.83307864 | 0.385083921 | 0.01094211 |
