# Supplementary material for: A Novel Signature of Necroptosis-Associated Genes as a Potential Prognostic Tool for Head and Neck Squamous Cell Carcinoma
Source: Front Genet. 2022 Jun 9;13:907985. doi: 10.3389/fgene.2022.907985 (PMC9218670; doi:10.3389/fgene.2022.907985)
Supplement: Supplementary file 3 [file Table3.DOCX]

Tables S3. GSEA report of pathways significantly enriched in high and low risk groups.

| **NAME** | **SIZE** | **ES** | **NES** | **NOM p-val** | **FDR q-val** |
| --- | --- | --- | --- | --- | --- |
| KEGG_BASAL_CELL_CARCINOMA | 55 | 0.60602933 | 1.9218568 | 0.001941748 | 0.36241376 |
| KEGG_GLYCOSAMINOGLYCAN_BIOSYNTHESIS_CHONDROITIN_SULFATE | 22 | 0.7568013 | 1.9100772 | 0.003960396 | 0.20684355 |
| KEGG_PENTOSE_PHOSPHATE_PATHWAY | 27 | 0.59933364 | 1.8113779 | 0.005964215 | 0.31478345 |
| KEGG_HEDGEHOG_SIGNALING_PATHWAY | 56 | 0.53046614 | 1.8011185 | 0.011494253 | 0.25415853 |
| KEGG_GLYCOSYLPHOSPHATIDYLINOSITOL_GPI_ANCHOR_BIOSYNTHESIS | 25 | 0.63080436 | 1.7852032 | 0.00929368 | 0.22905964 |
| KEGG_WNT_SIGNALING_PATHWAY | 150 | 0.46567485 | 1.744182 | 0.020715632 | 0.2606035 |
| KEGG_PENTOSE_AND_GLUCURONATE_INTERCONVERSIONS | 28 | 0.5973797 | 1.7308756 | 0.021276595 | 0.24322581 |
| KEGG_FOCAL_ADHESION | 199 | 0.51790047 | 1.7062249 | 0.036885247 | 0.2535227 |
| KEGG_ECM_RECEPTOR_INTERACTION | 84 | 0.57708174 | 1.6958095 | 0.058212057 | 0.24161182 |
| KEGG_RENAL_CELL_CARCINOMA | 70 | 0.46489683 | 1.6442232 | 0.01734104 | 0.295482 |
| KEGG_GLYCOLYSIS_GLUCONEOGENESIS | 62 | 0.4783958 | 1.632499 | 0.025242718 | 0.28814587 |
| KEGG_FRUCTOSE_AND_MANNOSE_METABOLISM | 33 | 0.51629305 | 1.5871547 | 0.049212597 | 0.3396336 |
| KEGG_GLYCOSAMINOGLYCAN_BIOSYNTHESIS_KERATAN_SULFATE | 15 | 0.6227197 | 1.5821882 | 0.046277665 | 0.32273033 |
| KEGG_STARCH_AND_SUCROSE_METABOLISM | 52 | 0.45259455 | 1.5785921 | 0.02739726 | 0.3056168 |
| KEGG_REGULATION_OF_ACTIN_CYTOSKELETON | 213 | 0.41413563 | 1.543799 | 0.04950495 | 0.3419818 |
| KEGG_ADHERENS_JUNCTION | 73 | 0.4597346 | 1.5284553 | 0.09596929 | 0.34534982 |
| KEGG_NOTCH_SIGNALING_PATHWAY | 47 | 0.451698 | 1.5187883 | 0.08971962 | 0.3420963 |
| KEGG_SMALL_CELL_LUNG_CANCER | 84 | 0.45931426 | 1.5155946 | 0.08846154 | 0.32789525 |
| KEGG_TGF_BETA_SIGNALING_PATHWAY | 85 | 0.43404886 | 1.5078869 | 0.08416834 | 0.32300034 |

| **NAME** | **SIZE** | **ES** | **NES** | **NOM p-val** | **FDR q-val** |
| --- | --- | --- | --- | --- | --- |
| KEGG_PRIMARY_IMMUNODEFICIENCY | 35 | -0.8551832 | -2.140327 | 0 | 0.017955266 |
| KEGG_ASTHMA | 28 | -0.8347725 | -2.0819173 | 0 | 0.018395558 |
| KEGG_AUTOIMMUNE_THYROID_DISEASE | 50 | -0.74769276 | -2.08062 | 0 | 0.012431461 |
| KEGG_INTESTINAL_IMMUNE_NETWORK_FOR_IGA_PRODUCTION | 46 | -0.7799718 | -2.0309203 | 0 | 0.019128727 |
| KEGG_TYPE_I_DIABETES_MELLITUS | 41 | -0.7821459 | -1.9788891 | 0.001923077 | 0.027242737 |
| KEGG_ALLOGRAFT_REJECTION | 35 | -0.851405 | -1.9597499 | 0 | 0.028405271 |
| KEGG_HEMATOPOIETIC_CELL_LINEAGE | 85 | -0.6430032 | -1.9568232 | 0.001945525 | 0.024879407 |
| KEGG_T_CELL_RECEPTOR_SIGNALING_PATHWAY | 108 | -0.5566765 | -1.9401574 | 0.007968128 | 0.025912447 |
| KEGG_NATURAL_KILLER_CELL_MEDIATED_CYTOTOXICITY | 132 | -0.5259088 | -1.9167242 | 0.005725191 | 0.029382363 |
| KEGG_GRAFT_VERSUS_HOST_DISEASE | 37 | -0.8226695 | -1.9162976 | 0.003759399 | 0.02670664 |
| KEGG_CYTOKINE_CYTOKINE_RECEPTOR_INTERACTION | 264 | -0.51869893 | -1.9146081 | 0.007905139 | 0.024902206 |
| KEGG_ANTIGEN_PROCESSING_AND_PRESENTATION | 81 | -0.60054123 | -1.8084649 | 0.027504912 | 0.059303056 |
| KEGG_B_CELL_RECEPTOR_SIGNALING_PATHWAY | 75 | -0.55603564 | -1.7806025 | 0.024793388 | 0.06831653 |
| KEGG_LINOLEIC_ACID_METABOLISM | 29 | -0.5761228 | -1.7708861 | 0.00610998 | 0.06807145 |
| KEGG_ARACHIDONIC_ACID_METABOLISM | 58 | -0.50130635 | -1.7578791 | 0.002028398 | 0.06993233 |
| KEGG_LEISHMANIA_INFECTION | 70 | -0.59395224 | -1.7207004 | 0.03807615 | 0.0846837 |
| KEGG_CELL_ADHESION_MOLECULES_CAMS | 131 | -0.5433893 | -1.7094867 | 0.046277665 | 0.08682568 |
| KEGG_JAK_STAT_SIGNALING_PATHWAY | 155 | -0.4324697 | -1.7020087 | 0.024 | 0.08609419 |
| KEGG_CHEMOKINE_SIGNALING_PATHWAY | 188 | -0.48847663 | -1.7010978 | 0.042168673 | 0.08191157 |
