# Supplementary material for: A Novel Signature of Necroptosis-Associated Genes as a Potential Prognostic Tool for Head and Neck Squamous Cell Carcinoma
Source: Front Genet. 2022 Jun 9;13:907985. doi: 10.3389/fgene.2022.907985 (PMC9218670; doi:10.3389/fgene.2022.907985)
Supplement: Supplementary file 4 [file Table4.DOCX]

Tables S4. The immune cells in risk groups .

| **immune** | **cor** | **pvalue** |
| --- | --- | --- |
| B cell_TIMER | -0.333382257 | 6.35E-14 |
| T cell CD4+_TIMER | -0.12763636 | 0.005101652 |
| T cell CD8+_TIMER | -0.190590309 | 2.63E-05 |
| Neutrophil_TIMER | -0.177882282 | 9.14E-05 |
| Myeloid dendritic cell_TIMER | -0.132650315 | 0.003619906 |
| B cell naive_CIBERSORT | -0.132406726 | 0.00365932 |
| B cell memory_CIBERSORT | -0.251523724 | 2.32E-08 |
| T cell CD8+_CIBERSORT | -0.376281824 | 1.36E-17 |
| T cell CD4+ naive_CIBERSORT | 0.135486344 | 0.002936161 |
| T cell CD4+ memory resting_CIBERSORT | 0.11017405 | 0.015741496 |
| T cell CD4+ memory activated_CIBERSORT | -0.095267952 | 0.036931364 |
| T cell follicular helper_CIBERSORT | -0.304742027 | 8.97E-12 |
| T cell regulatory (Tregs)_CIBERSORT | -0.271953346 | 1.39E-09 |
| T cell gamma delta_CIBERSORT | -0.140049089 | 0.002101555 |
| NK cell resting_CIBERSORT | 0.164618444 | 0.000292396 |
| Macrophage M0_CIBERSORT | 0.213121208 | 2.46E-06 |
| Macrophage M1_CIBERSORT | -0.229371749 | 3.77E-07 |
| Myeloid dendritic cell activated_CIBERSORT | 0.112786713 | 0.013417767 |
| Mast cell activated_CIBERSORT | -0.184109811 | 4.95E-05 |
| Mast cell resting_CIBERSORT | 0.2208348 | 1.03E-06 |
| Neutrophil_CIBERSORT | -0.113208559 | 0.013072372 |
| B cell naive_CIBERSORT-ABS | -0.163831598 | 0.000312881 |
| B cell memory_CIBERSORT-ABS | -0.258307348 | 9.34E-09 |
| B cell plasma_CIBERSORT-ABS | -0.117575844 | 0.009931953 |
| T cell CD8+_CIBERSORT-ABS | -0.422833484 | 3.07E-22 |
| T cell CD4+ naive_CIBERSORT-ABS | 0.135403792 | 0.002953713 |
| T cell CD4+ memory activated_CIBERSORT-ABS | -0.12178379 | 0.007559376 |
| T cell follicular helper_CIBERSORT-ABS | -0.422303446 | 3.50E-22 |
| T cell regulatory (Tregs)_CIBERSORT-ABS | -0.346408171 | 5.59E-15 |
| T cell gamma delta_CIBERSORT-ABS | -0.140228778 | 0.00207364 |
| NK cell resting_CIBERSORT-ABS | 0.125782748 | 0.005788201 |
| NK cell activated_CIBERSORT-ABS | -0.144562533 | 0.001495037 |
| Macrophage M1_CIBERSORT-ABS | -0.32628573 | 2.28E-13 |
| Macrophage M2_CIBERSORT-ABS | -0.170448966 | 0.000175356 |
| Myeloid dendritic cell resting_CIBERSORT-ABS | -0.127683539 | 0.005085178 |
| Mast cell activated_CIBERSORT-ABS | -0.218283987 | 1.38E-06 |
| Mast cell resting_CIBERSORT-ABS | 0.142062963 | 0.001807481 |
| Neutrophil_CIBERSORT-ABS | -0.145080171 | 0.001436883 |
| B cell_QUANTISEQ | -0.283292525 | 2.61E-10 |
| Monocyte_QUANTISEQ | -0.103848286 | 0.02288044 |
| Neutrophil_QUANTISEQ | -0.097612875 | 0.032506738 |
| NK cell_QUANTISEQ | 0.126096773 | 0.005666344 |
| T cell CD4+ (non-regulatory)_QUANTISEQ | 0.142784811 | 0.001711607 |
| T cell CD8+_QUANTISEQ | -0.35570566 | 9.20E-16 |
| T cell regulatory (Tregs)_QUANTISEQ | -0.264937148 | 3.75E-09 |
| T cell_MCPCOUNTER | -0.360467602 | 3.63E-16 |
| T cell CD8+_MCPCOUNTER | -0.37945851 | 6.92E-18 |
| cytotoxicity score_MCPCOUNTER | -0.11033468 | 0.015626454 |
| NK cell_MCPCOUNTER | -0.198155048 | 1.27E-05 |
| B cell_MCPCOUNTER | -0.329070005 | 1.87E-13 |
| Monocyte_MCPCOUNTER | -0.099409069 | 0.029467215 |
| Macrophage/Monocyte_MCPCOUNTER | -0.099409069 | 0.029467215 |
| Myeloid dendritic cell_MCPCOUNTER | -0.281534534 | 4.06E-10 |
| Neutrophil_MCPCOUNTER | 0.09756314 | 0.032630364 |
| Cancer associated fibroblast_MCPCOUNTER | 0.166539568 | 0.000252192 |
| Myeloid dendritic cell activated_XCELL | -0.323359673 | 3.81E-13 |
| B cell_XCELL | -0.427869323 | 8.68E-23 |
| T cell CD4+ naive_XCELL | -0.285540116 | 1.86E-10 |
| T cell CD4+ central memory_XCELL | -0.271364293 | 1.51E-09 |
| T cell CD8+ naive_XCELL | -0.116269747 | 0.010792314 |
| T cell CD8+_XCELL | -0.381961096 | 4.04E-18 |
| T cell CD8+ central memory_XCELL | -0.372427817 | 3.07E-17 |
| T cell CD8+ effector memory_XCELL | -0.191063282 | 2.51E-05 |
| Class-switched memory B cell_XCELL | -0.315358795 | 1.52E-12 |
| Common lymphoid progenitor_XCELL | 0.151356264 | 0.000879163 |
| Common myeloid progenitor_XCELL | -0.10159905 | 0.026022051 |
| Myeloid dendritic cell_XCELL | -0.251994877 | 2.18E-08 |
| Endothelial cell_XCELL | -0.169696362 | 0.000187497 |
| Cancer associated fibroblast_XCELL | -0.122236777 | 0.007336915 |
| Granulocyte-monocyte progenitor_XCELL | -0.120184269 | 0.008393913 |
| Hematopoietic stem cell_XCELL | -0.238257344 | 1.27E-07 |
| Macrophage M1_XCELL | -0.162273086 | 0.000357469 |
| Macrophage M2_XCELL | -0.115876655 | 0.011063874 |
| Mast cell_XCELL | -0.181942907 | 6.09E-05 |
| B cell memory_XCELL | -0.346274871 | 5.74E-15 |
| Monocyte_XCELL | -0.155249788 | 0.000642046 |
| B cell naive_XCELL | -0.125052419 | 0.006080759 |
| Plasmacytoid dendritic cell_XCELL | -0.383729868 | 2.75E-18 |
| B cell plasma_XCELL | -0.227209027 | 4.88E-07 |
| T cell gamma delta_XCELL | -0.235213594 | 1.86E-07 |
| T cell CD4+ Th2_XCELL | 0.111035421 | 0.01497631 |
| T cell regulatory (Tregs)_XCELL | -0.095227954 | 0.037011066 |
| immune score_XCELL | -0.41811553 | 0 |
| stroma score_XCELL | -0.14443977 | 0.001509142 |
| microenvironment score_XCELL | -0.385925178 | 0 |
| B cell_EPIC | -0.331130777 | 1.29E-13 |
| Cancer associated fibroblast_EPIC | 0.169387888 | 0.000196623 |
| T cell CD4+_EPIC | 0.155989935 | 0.000612753 |
| T cell CD8+_EPIC | -0.299679144 | 2.56E-11 |
| Endothelial cell_EPIC | -0.214364863 | 2.27E-06 |
| Macrophage_EPIC | -0.100910486 | 0.027092035 |
